# A novel long non-coding RNA-based prognostic signature for renal cell carcinoma patients with stage IV and histological grade G4

**DOI:** 10.1080/21655979.2021.1971022

**Published:** 2021-09-09

**Authors:** Ning Li, Haiying Zhang, Keyao Hu, Jianfeng Chu

**Affiliations:** Department of Urology, Yantaishan Hospital, Yantai, Shandong , P.R. China

**Keywords:** RCC, prognosis, survival, lncRNA signature

## Abstract

This study aimed to establish a lncRNA-based signature for predicting the prognosis of patients with high stage and grade renal cell carcinoma (RCC). According to the Surveillance, Epidemiology, and End Results (SEER) database, sex, age, grade, stage, surgery, chemotherapy, radiation, tumor size, and marital status were the independent prognostic factors for RCC and also had significant correlations with the overall survival through Cox univariate and multivariate analyses. Noticeably, among these influencing factors, the histological classification of undifferentiated group and pathological stage IV had the greatest prognostic risks for RCC patients. Furthermore, based on the samples at stage IV and histological grade G4 from The Cancer Genome Atlas (TCGA) portal, 9 key lncRNAs, including KIAA2012, CCNT2-AS1, ITPKB-AS1, TBX2-AS1, NUTM2A-AS1, LINC02522, LINC02384, LINC01559, and LINC00865 were identified and a prognostic signature was constructed by Lasso analysis and Cox regression model. The Kaplan-Meier analysis suggested that patients at stage IV and histological grade of G4 in high risk score group had a worse overall survival than that in low risk score group. The following receiver operating characteristic curve (ROC) curves also showed that this signature possesses a better predictive power performance. Pathway enrichment analysis discovered that 9 lncRNAs held potential roles in cell division, cell cycle, DNA damage and cytokines levels in RCC. This work indicates that the established 9-lncRNA signature has a good capacity in predicting the prognosis of RCC patients with stage IV and histological grade of G4, and may be helpful for guiding the treatment strategies for RCC patients.

## Introduction

Renal cell carcinoma (RCC) is recognized as a common and deadly disease with a worldwide estimate of ~400,000 new cases and ~175,000 deaths [1]. Despite progressions in cancer control and survival improvement, locally advanced disease and distant metastases are still diagnosed in a notable proportion of patients, leading to its treatment remaining a challenge for oncologists [2]. Currently, surgery is considered to be the most important and effective method for treating the early stage of RCC [3]. However, 20%~30% patients still have recurrence or distant metastasis after surgery [4]. Additionally, RCC is not sensitive to chemotherapy or radiotherapy, resulting in the un-satisfactorily clinical outcomes for patients. Hence, identifying usefully prognostic biomarkers is of great significance for ameliorating the survival benefits of RCC patients.

Long non-coding RNA (lncRNA) refers to RNA transcripts ≥200 bp that are not equipped with protein-coding capacity [5]. Accumulating evidence demonstrates that lncRNAs play extensive regulatory roles in a variety of cellular processes, including transcriptional and post-transcriptional regulation [6,7]. Due to their strong tissue specific, they are potentially effective biomarkers for the survival of numerous cancers [8]. Identification of specific lncRNAs biomarkers may therefore be of clinical significance for the prognosis of RCC patients. Indeed, numerous lncRNAs have been identified to be related to the progression and prognosis of RCC. Qu et al. constructed a 4-lncRNA signature, including ENSG00000255774, ENSG00000248323, ENSG00000260911, and ENSG00000231666 to improve postoperative risk stratification for patients with localized clear cell RCC using the LASSO Cox regression model [9]. Zhang et al. established an 11-lncRNA signature that was clearly associated with the overall survival rates in clear cell RCC using the data from The Cancer Genome Atlas (TCGA) database [10]. At present, studies on the mechanism and function of lncRNAs in RCC are still under exploration. Therefore, identification of more effective lncRNA signatures undoubtedly provides more possible references for the clinical study of RCC.

In our work, to discriminate the sinnvoll prognostic factors, a large number data of RCC patients were retrieved from the Surveillance, Epidemiology, and End Results (SEER) database to analyze the significant prognostic factors. Moreover, to further ascertain the specific lncRNAs biomarkers, the RNA sequencing data of 38 tumor samples of RCC with the greatest prognostic factors of stage IV and histological grade G4 and 128 adjacent normal samples were extracted to construct a high stage and grade-related lncRNA signature associated with the prognosis of RCC patients. Ulteriorly, to explore the underlying mechanism behind this signature modulating the progression of RCC, pathway enrichment analyses were applied to identify cell cycle and cell division-related signaling. The established lncRNA signature may serve as a potential prognosticator the RCC, holding promise for acting as a potential therapeutic target for the therapy of RCC.

## Materials and methods

### Data source from SEER

SEER database is programmed by US National Cancer Institute, which collects, processes, and provides data on approximately 10% of the US population [11]. Data of the present study were obtained from SEER database using the SEER*Stat software program (version 8.3.3) under a user agreement. The patients’ demographic variables such as age of diagnosis, gender, marry, insure, surgery, chemotherapy, radiation, laterality, tumor size, grade, stage, race, histology, survival months, and vital status recode were extracted. Furthermore, we also excluded patients with missing information on the survival time/status, chemoradiotherapy information, or on the tumor stage.

### Acquisition of gene expression data of RCC from TCGA database

The RNA-Seq expression data and the corresponding clinical data were downloaded from TCGA-KICH, TCGA-KIRC, and TCGA-KIRP datasets [12]. A total of 38 RCC patients and 128 normal patients were screened with pathological stage IV and histological grade of G4.

### Construction of a prognostic signature for high stage and grade patients

Using the Cox univariate regression analysis, the survival-associated lncRNAs were identified. Next, Lasso regression analysis was used to obtain the most strongly survival-associated lncRNAs in RCC, further fitting in Cox multivariate regression analysis to generate the regression coefficient for each lncRNA. The following formula based on a combination of Cox coefficient and the expression of lncRNA was used to calculate the risk score: Risk score = (coefficient _lncRNA1_ × lncRNA1 expression) + (coefficient _lncRNA2_ × lncRNA2 expression) + … + (coefficient _lncRNAn_ ×lncRNAn expression). The median risk score was used as a cutoff to divide the RCC patients into high- and low-risk groups. The receiver operating characteristic (ROC) curves were plotted and the area under the curve (AUC) values were calculated with ‘SurvivalROC’ R package, which was used to evaluate the predictive power [13].

### Gene set enrichment analysis (GSEA)

GSEA was conducted between the high and low-risk score groups to explore the relationship between risk score and lncRNA function. GSEA version 4.0.3 software (Cambridge, MA, USA) was used to perform the GSEA, and p values <0.05 with false discovery rate (FDR) <0.05 were considered statistically significant [14].

### Pathway enticement for protein–protein interaction (PPI) network of co-expressed genes of 9-lncRNAs

The co-expressed genes of nine lncRNAs were analyzed through calculating Pearson correlation values between the nine lncRNAs and 854 differentially genes RCC and normal patients. Co-expressed genes were screened out according to Pearson coefficient greater than 0.4 and P less than 0.01 and were performed to establish protein–protein interaction (PPI) network by STRING dataset [15] and the most important module was identified by MCODE plugin in Cytoscape software [16]. The key genes contained in key module were incorporated to perform pathway enrichment analysis by metascape [17,18].

### Statistical analyses

All the data were analyzed using the SPSS 22.0 software (IBM SPSS, Armonk, NY, USA). Overall survival curves were plotted using the Kaplan–Meier approach. Cox proportional hazards model was used to determine the influences of the collected clinicopathological factors on the overall survival or disease-specific survival of RCC patients. Differences in patients’ characteristics were compared by student t test for continuous variables and chi-square analysis for categorical variables. A two-tailed P value less than 0.05 was considered as statistical significance.

## Results

In this current study, we intended to uncover the significantly independent prognosticators for RCC and to construct a lncRNA-based signature for patients with these distinguished features. Clinical characteristics of RCC patients were incorporated into analyzing the independent prognostic factors for RCC. In accordance with the significant value, the greatest prognosticators were identified, combined with RNA sequence, and thus the prognostic-associated lncRNA signature was built. Enrichment analysis was performed to explore the underlying mechanism for RCC progression.

### Demographics of patients

A total of 27,435 individuals with a diagnosis of RCC were identified according to the SEER database. The clinical characteristics of these RCC patients are summarized in Table 1. In this population, the mean age of RCC patients was 60.1 ± 12.3 years. Most of them were white, male, married, and with insure. Histologically, adenocarcinoma was the main histologic type of RCC, accounting for 98.8%. Approximately 64.4% of patients were in stage I, and 96.5% underwent surgery, however, few patients received chemotherapy and radiation, which account for 6.5% and 2.6%, respectively.

**Table 1.** Clinical characteristics of RCC patients included in this study according to the SEER database

variables	variables	n(%)
age(years)		60.1 ± 12.3
	<60	12,618(46)
	≥60	14,817(54)
race	black	3151(11.5)
	white	22,365(81.5)
	other	1919(7)
sex	female	9941(36.2)
	male	17,494(63.8)
histology		
	Epithelialneoplasms	72(0.3)
	squamous cell neoplams	43(0.2)
	transitional cell papillomas	
and carcinomas	188(0.7)	
	adenomas and adenocarcinomas	37,104(98.8)
	cystic, mucinous and serous neoplams	18(0.1)
	ductal and lobular neoplams	10(0.04)
grade		
	I	2992(10.9)
	II	13,763(50.2)
	III	8300(30.3)
	IV	2380(8.7)
stage		
	I	17,715(64.6)
	II	2511(9.2)
	III	4702(17.1)
	IV	2507(9.1)
surgery		
	yes	26,474(96.5)
	no	961(3.5)
chemotherapy		
	yes	1775(6.5)
	no	25,660(93.5)
radiation		
	yes	715(2.6)
	no	26,720(97.4)
tumor size		
	≤5 cm	16,178(59)
	>5 cm	11,149(40.6)
	unknown	108(0.4)
insure		
	yes	26,680(97.2)
	no	755(2.8)
marry		
	yes	17,559(64%)
	no	9876(36%)
laterality		
	left	13,387(48.8)
	right	14,017(51.1)
	other	31(0.1)

### Identification of independent prognosticator for RCC based on SEER database

**Figure 1.**
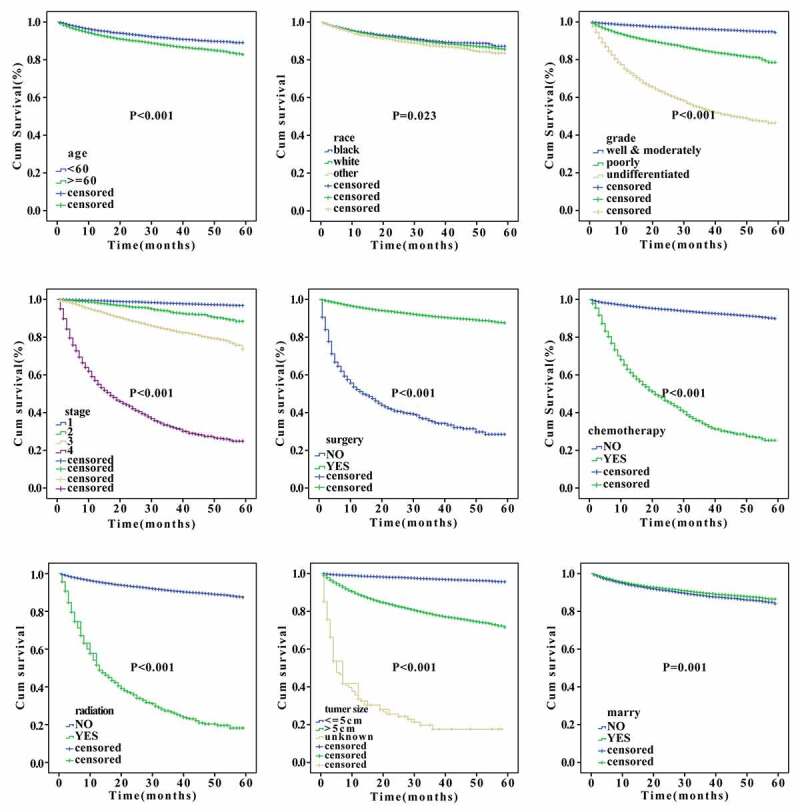
Kaplan-Meier analysis of prognostic factors according to the SEER database

Cox univariate and multivariate analyses were used to identify the independent predictive factors affecting the overall survival of RCC. We found that sex, age, grade, stage, surgery, chemotherapy, radiation, tumor size, and marital status can all be used as independent prognostic factors for the overall survival of RCC (p < 0.05, Table 2). Noticeably, among other influencing factors, the histological classification of undifferentiated group and pathological stage IV had the greatest prognostic risks, with the hazard ratio (HR) values of 3.05 and 6.515, respectively. Furthermore, Cox univariate and multivariate analyses for specific survival were also performed. Results shown in Table 3 indicated that age, grade, stage, surgery, chemotherapy, radiation, tumor size, and marital status can be used as independent prognostic factors for disease-specific survival of RCC (p < 0.05, Table 3). Importantly, the hazard ratio (HR) of patients who underwent surgery was 0.254 times that of those who did not, suggesting that surgery can significantly reduce patients’ risk. However, the HR values of patients who received radiotherapy and chemotherapy were much higher than those who did not receive radiotherapy and chemotherapy (13.858 and 13.7058 times, respectively), indicating that radiotherapy and chemotherapy might lead to a worse prognosis. Besides, we analyzed the effects of the clinical parameters on the overall survival of patients of RCC using Kaplan–Meier approach. The results showed that age, race, grade, stage, surgery, chemotherapy, radiation, tumor size, and marital status had significant actions on the survival (p < 0.05, Figure 1).

**Table 2.** Cox univariate and multivariate analysis for the overall survival of patients with RCC from SEER database

Variables		Univariate		Multivariate	
		HR(95%CI)	P value	HR(95%CI)	P value
sex	female	1		1	
	male	1.24(1.158–1.328)	<0.001	1.106(1.031–1.187)	0.005
age	<60	1		1	
	≥60	1.856(1.734–1.987)	<0.001	1.708(1.594–1.829)	<0.001
race	black	1		1	
	white	0.983(0.89–1.086)	0.732		
	other	1.11(0.957–1.287)	0.169		
Grade	well&moderately differentiated	1		1	
	poorly differentiated	2.539(2.356–2.737)	<0.001	1.61(1.487–1.742)	<0.001
	undifferentiated	8.078(7.442–8.768)	<0.001	3.05(2.78–3.346)	<0.001
stage	I	1		1	
	II	1.714(1.492–1.969)	<0.001	1.098(0.94–1.283)	0.237
	III	3.577(3.27–3.912)	<0.001	2.027(1.822–2.255)	<0.001
	IV	20.037(18.515–21.684)	<0.001	6.515(5.763–7.365)	<0.001
surgery	no	1		1	
	yes	0.095(0.087–0.103)	<0.001	0.263(0.238–0.292)	<0.001
chemotherapy	no	1		1	
	yes	9.116(8.493–9.783)	<0.001	1.139(1.037–1.251)	0.006
radiation	no	1		1	
	yes	10.043(9.143–11.032)	<0.001	1.402(1.263–1.556)	<0.001
tumor size	≤5 cm	1		1	
	>5 cm	4.086(3.805–4.388)	<0.001	1.524(1.388–1.673)	<0.001
	unknown	29.792(23.87–37.182)	<0.001	1.812(1.417–2.381)	<0.001
insure	yes	1		1	
	no	1.065(0.882–1.287)	0.512		
marital status	no	1		1	
	yes	0.766(0.718–0.817)	0.001	0.72(0.674–0.769)	<0.001
laterality	left	1		1	
	right	0.919(0.862–0.980)	0.010	0.955(0.895–1.017)	0.153
	other	7.490(4.821–11.639)	<0.001	0.999(0.633–1.579)	0.998

**Table 3.** Cox univariate and multivariate analysis for the specific survival of patients with RCC from SEER database

Variables		Univariate		Multivariate	
		HR(95%CI)	P value	HR(95%CI)	P valuer
sex	female	1		1	
	male	1.247(1.149–1.354)	<0.001	1.013(0.931–1.101)	0.768
age	<60	1		1	
	≥60	1.517(1.401–1.641)	<0.001	1.387(1.28–1.503)	<0.001
race	black	1		1	
	white	1.092(0.965–1.237)	0.162	0.806(0.710–0.915)	0.001
	other	1.281(1.072–1.532)	0.006	0.836(0.698–1.001)	0.051
Grade	well&moderately differentiated	1		1	
	poorly differentiated	4.283(3.871–4.738)	<0.001	2.162(1.946–2.401)	<0.001
	undifferentiated	15.992(14.412–17.745)	<0.001	4.187(3.736–4.692)	<0.001
stage	I	1		1	
	II	3.328(2.736–4.047)	<0.001	1.74(1.403–2.158)	<0.001
	III	8.62(7.539–9.856)	<0.001	4.052(3.472–4.73)	<0.001
	IV	58.901(52.129–66.551)	<0.001	14.723(12.474–17.378)	<0.001
surgery	no	1		1	
	yes	0.077(0.07–0.085)	<0.001	0.254(0.227–0.285)	<0.001
chemotherapy	no	1		1	
	yes	13.705(12.666–14.828)		1.156(1.047–1.277)	0.004
radiation	no	1		1	
	yes	13.858(12.551–15.301)	<0.001	1.463(1.312–1.630)	<0.001
tumor size	≤5 cm	1		1	
	>5 cm	8.318(7.491–9.237)	<0.001	1.838(1.619–2.087)	<0.001
	unknown	68.019(53.573–86.36)	<0.001	2.255(1.73–2.941)	<0.001
insure	yes	1		1	
	no	1.238(1.002–1.529)	0.048	1.019(0.822–1.263)	0.863
marital status	no	1		1	
	yes	0.879(0.813–0.951)	0.001	0.837(0.772–0.908)	<0.001
laterality	left	1		1	
	right	0.901(0.835–0.973)	0.008	0.949(0.879–1.024)	0.176
	other	8.073(4.931–13.218)	<0.001	0.876(0.526–1.461)	0.613

### Construction of a prognostic-associated lncRNA signature in RCC

Analysis of the SEER database indicated that undifferentiated histological grade and pathological stage IV had the greatest prognostic risk for RCC patients. Thus, in order to explore the relevant key molecules, we extracted samples of RCC with pathological stage IV and histological grade of G4 from the TCGA database for analysis.

**Figure 2.**
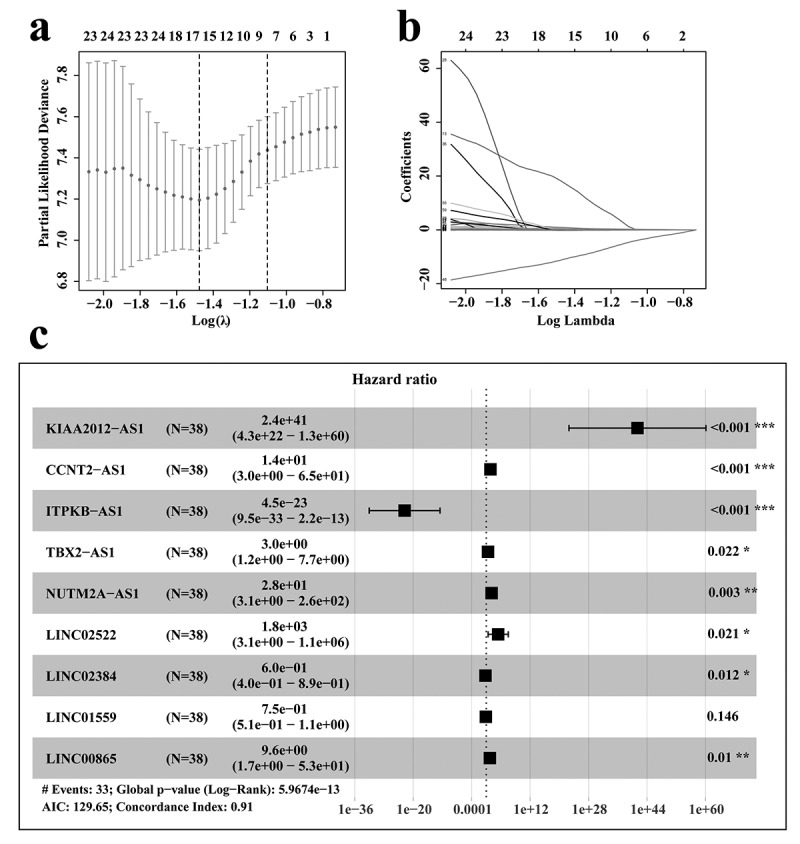
Selection of prognosis-related lncRNAs in RCC patients with stage IV and histological grade G4

According to the RNA sequencing expression files of RCC cases from TCGA database, we obtained all expressions of lncRNAs associated with the stage IV and histological grade of G4. These lncRNAs were then analyzed by Cox univariate analysis to identify prognosis-related lncRNAs. To further screen out the key lncRNAs for RCC patients with stage IV and histological grade of G4, Lasso regression and Cox multivariate analyses were performed (Figure 2a-c). The results showed that nine lncRNAs, including KIAA2012, CCNT2-AS1, ITPKB-AS1, TBX2-AS1, NUTM2A-AS1, LINC02522, LINC02384, LINC01559, and LINC00865, were selected to construct the prognostic model.

(a-b) Lasso regression analysis. (c) Cox multivariate analysis to select the key lncRNAs most relevant to prognosis.

**Figure 3.**
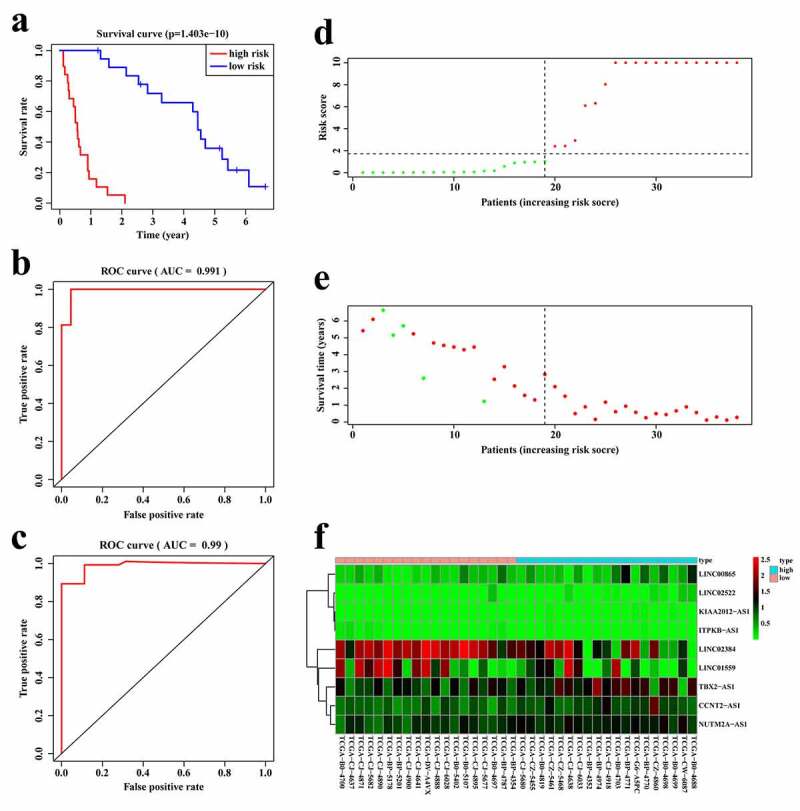
**Establishment of the 9-lncRNA prognostic signature based on the TCGA cohort**. (a) Kaplan-Meier analysis for patients in high and low risk score groups. (b) ROC curve for 1 year overall survival prediction of the 9-lncRNA signature. (c) ROC curve for 2 year overall survival prediction of the 9-lncRNA signature. (d) Distribution of risk score. (e) Distribution of survival time. (f) Heat map of the 9 lncRNAs expression

According to the expression of these nine lncRNAs for the overall survival prediction of RCC patients with stage IV and histological grade of G4, we established a risk score of the 9-lncRNA signature with the following formula: Risk score = (KIAA2012-AS1 × 95.28813027) + (CCNT2-AS1 × 2.637995925)+ (ITPKB-AS1×-51.44559677)+ (TBX2-AS1 × 1.102500797)+ (NUTM2A-AS1 × 3.340712729)+ (LINC02522 × 7.500647683)+ (LINC02384×-0.515463704)+ (LINC01559×-0.284528539)+ (LINC00865 × 2.265026437). We further validated the predictive power and stability of the 9-lncRNA signature in predicting the overall survival of RCC patients with stage IV and histological grade of G4 based on the TCGA cohort. Patients were divided into high- and low-risk score groups according to the median score. The Kaplan–Meier survival curve revealed that the survival time of the low-risk score group was significantly higher than that of the high-risk group (p < 0.01, Figure 3a). Moreover, the time-dependent ROC curves of the 9-lncRNA prognostic signature in the first two years were plotted. Surprisingly, the AUC of the 9-lncRNA prognostic signature risk score in the first and second year was 0.991 (Figure 3b) and 0.99 (Figure 3c), respectively, indicating that this signature had a powerful capacity in predicting the overall survival of RCC patients with stage IV and histological grade of G4. The distribution of the risk scores and survival status of each patients were shown in Figure 3d, e, and demonstrated that as the risk score increased, the number of patient deaths also increased. In addition, the heatmap of the expressions levels of nine lncRNAs was presented in Figure 3f, and revealed that patients with high-risk score tended to have high expressions of risk lncRNAs, and patients with low-risk score tended to have high expressions of protective lncRNAs.

### Pathway enrichment for nine-lncRNA signature

**Figure 4.**
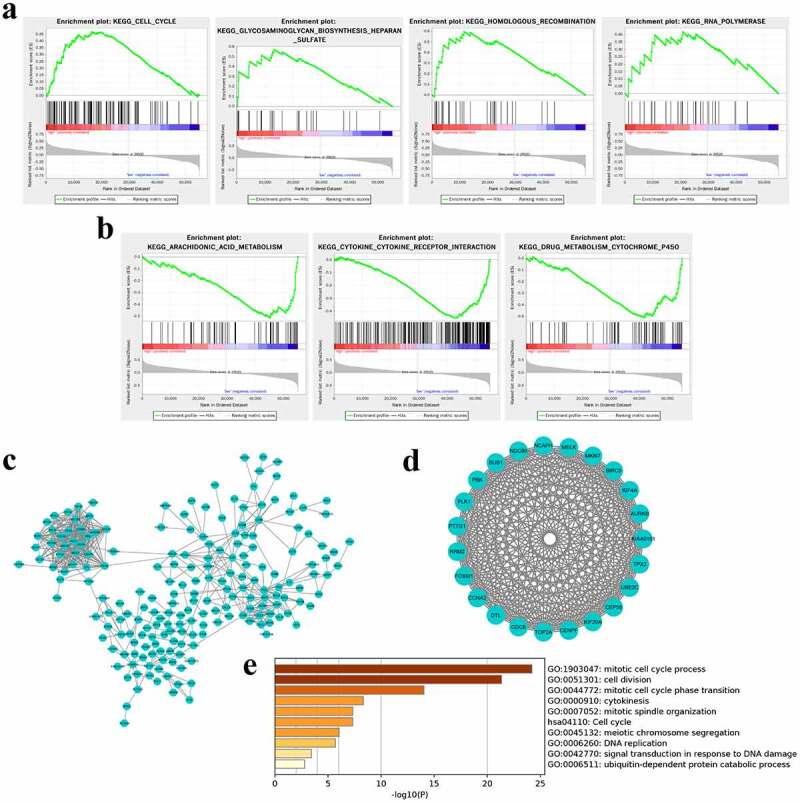
**Pathway enrichment analyses by GSEA and PPI/metascape**. (a) Signaling pathways were positively correlated with the risk score. (b) Signaling pathways were negatively correlated with the risk score. (c) The coexpressed genes of nine lncRNAs were used to build PPI network by STRING. (d) The most important module including key genes was identified by the MCODE plugin in Cytoscape. (e) Key genes mediating the crucial pathways were enriched by metascape

To investigate the signaling pathways associated with the risk score signature, GSEA was conducted between high and low-risk groups based on the TCGA cohort. The results showed that GLYCOSAMINOGLYCAN_BIOSYNTHESIS_HEPARAN_SULFATE, RNA_POLYMERASE, HOMOLOGOUS_RECOMBINATION, CELL_CYCLE were positively correlated with the risk score (Figure 4a). In the low-risk score groups, the enriched pathways focused on the KEGG_ARACHIDONIC_ACID_METABOLISM, KEGG_CYTOKINE_CYTOKINE_RECEPTOR_INTERACTION, KEGG_DRUG_METABOLISM_CYTOCHROME_P450 (Figure 4b). These enriched pathways implied that the 9-lncRNA signature was correlated with the progression of RCC.

To further clarify the mechanism of this signature regulating the progression of RCC, 408 co-expressed genes of the nine lncRNAs were filtered and minimum required interaction score greater than 0.7 were executed to build PPI network by STRING (Figure 4c). By using the MCODE plugin in Cytoscape software, the most important module comprising 23 genes was identified (Figure 4d). These key gene were enriched to crucial signaling pathway though metascape and were significantly enriched in cell division and cell cycle, cytokines, and DNA damage, embodying in mitotic cell cycle process and cell cycle-phase transition, cell division, mitotic spindle organization, cell cycle, cytokines, meiotic chromosome segregation, DNA replication, signal transduction in response to DNA damage and so on (Figure 4e). These data indicated that the nine lncRNAs might exert vital roles in participating in progression of RCC through regulating cell division, cell cycle, DNA damage, and cytokines levels.

## Discussion

RCC is one of the most common malignancies of the urinary system. It originates from the urinary tubular epithelial system of renal parenchyma and accounts for 80%–90% of renal malignancies [18]. Recently, due to smoking, changes in diet and other reasons, the incidence of RCC has gradually increased [2]. Despite the improvements in RCC diagnosis and management observed during the last two decades, the survival of RCC is still unsatisfactory. Herein, we summarized the clinical factors of RCC and identifying the predictive prognosticator, which were conducive to improving the treatment of such diseases. Besides, an accurate prognostic signature was constructed by this study, which will help clinicians to make better and more objective judgment on the prognosis of RCC patients.

Initial analysis of RCC data from SEER database revealed that sex, age, grade, stage, surgery, chemotherapy, radiation, tumor size, and marital status were the independent prognostic factors for RCC patients. In addition, we also found that high and low levels of these factors were significantly correlated with survivals of patients with RCC, suggesting that these factors play important roles in clinician’s prediction of patient survival. Moreover, because RCC itself was not sensitive to radiotherapy and chemotherapy, thus few patients received the two treatment approaches. We also noted that tumor grading and staging were significantly associated with the prognosis, supporting the previous literature [19]. Furthermore, the histological classification of undifferentiated group and pathological stage IV had the greatest prognostic risks, hinting us that constructing a high stage and grade-related model would play a positive guiding role in the prognosis for RCC patients.

Prognostic evaluation remains necessary for the selection of appropriate treatments for RCC patients because of poor prognosis [20]. Some lncRNAs and lncRNA signatures have been considered as prognostic indicator in a variety of cancers. In RCC, although numerous lncRNA signatures were constructed to be associated with the prognosis [21–23], few of them correlated an lncRNA signature with the clinical outcome in the high stage and grade of RCC. In present study, pathological stage IV and histological grade of G4 associated lncRNAs were obtained according to the TCGA cohort. By Lasso analysis and Cox univariate and multivariate analysis, finally, a 9-lncRNA signature, including, KIAA2012, CCNT2-AS1, ITPKB-AS1, TBX2-AS1, NUTM2A-AS1, LINC02522, LINC02384, LINC01559, and LINC00865, was constructed with the potential to predict the prognosis for patients with high stage and grade of RCC. Thus, the risk score model was established based on the signature of these nine lncRNAs. What’s more, the Kaplan–Meier survival curve showed that patients in high-risk score group had a worse overall survival than that in low-risk score group. ROC curves also indicated that this 9-lncRNA signature may be served as a better prognostic biomarker. All these results confirmed the predictive power of the prognostic signature for patients with high stage and grade of RCC.

Among the nine lncRNAs, LINC01559 was reported as a member of a 6-lncRNA signature that is an noninvasive biomarker of RCC [24]. NUTM2A-AS1 was found to be highly expressed and linked to the prognosis and prognosis in gastric cancer, non-small cell lung carcinoma, hepatocellular carcinoma, suggesting its potential role in other cancers [25–27]. These reports pointed the important roles of LINC01559 and NUTM2A-AS1 in tumor progression and are consistent with our present results. The remaining lncRNAs are poorly reported in tumors and deserves to be analyzed further.

Regarding the results of GSEA, we found that several pathways, such as cell cycle, glycosaminoglycan biosynthesis heparin sulfate, homologous recombination, RNA polymerase were positively associated with risk scores whereas arachidonic acid metabolism, cytokine receptor interaction, drug metabolism cytochrome P450 were negatively correlated with risk scores. In addition, PPI and metascape analyses found that cell cycle, cell division, DNA damage, and cytokines levels were enriched by analyzing the pathway enrichment of nine lncRNAs-coexpressed genes. Cell cycle is one of the most important regulatory mechanisms of cellular growth and proliferation. Generally, dysregulation of this pathway is thought to be the first step in carcinogenesis of RCC [28]. A previous study also revealed that glycosaminoglycans may serve as potential biomarkers in RCC [29]. Besides, the metabolism of arachidonic acid by either the cyclooxygenase or lipoxygenase pathway is believed to play an important role in tumor promotion [30]. Microtubule Interacting and Trafficking Domain containing one has been reported to participate in cytokinesis of cell division, and high level of it has been found to trigger a worse survival of RCC, correlate with CD8 + T cell and mediate immune response and cytokine–cytokine receptor signalings [31]. Joon Chae et al. have determined that alteration in the DNA damage response pathway correlates with increased recurrence in locally advanced clear-cell RCC, hinting exploration of therapeutic agents targeting this pathway for treating these patients [32]. Therefore, the activation of the above mechanisms and pathways may lead to tumorigenesis and progression of RCC. These data further emphasized the importance of the identified lncRNAs in the present study.

However, there were some limitations to the present study. Firstly, incorporated cases were a small number that were insufficient for establishing an ideal signature model for predicting survival in patients with pathological stage IV and histological grade of G4 of RCC. Secondly, this signature is warranted to be verified in the further study through our institute collected cases or experimental analyses in vitro or in vivo. Further, mechanism of action behind this signature regulating RCC progression will be worth depth exploring.

## Conclusion

Taken together, the present study analyzed the effect of clinical features on the survival of RCC based on a large population samples that obtained from SEER database. Furthermore, a 9-lncRNA signature for predicting survival in patients with pathological stage IV and histological grade of G4 of RCC was constructed by analyzing the RNA-Seq expression profile in TCGA cohort. The risk score model was a potential prognostic biomarker for patients with high stage and grade of RCC. The results of pathway enrichment reflecting cell division and cell cycle-related signaling also deciphered the potential mechanism of its regulating the progression of RCC. Our results may provide a good predictive performance in the further treatment of RCC with pathological stage IV and histological grade of G4.

## Highlights

1. A nine-lncRNA signature was built to predict survival in patients with pathological stage IV and histological grade of G4 of RCC.

2. Independent prognosticators were identified to provide favorable evidence for clinical prediction.

3. Discriminating prognostic-associated lncRNA signature and enrichment analysis contributes to uncover the pathogenesis for RCC patients with stage IV and grade of G4.
